# Measurement of dynamic task related functional networks using MEG

**DOI:** 10.1016/j.neuroimage.2016.08.061

**Published:** 2017-02-01

**Authors:** George C. O’Neill, Prejaas K. Tewarie, Giles L. Colclough, Lauren E. Gascoyne, Benjamin A.E. Hunt, Peter G. Morris, Mark W. Woolrich, Matthew J. Brookes

**Affiliations:** a Sir Peter Mansfield Imaging Centre, School of Physics and Astronomy, University of Nottingham, University Park, Nottingham, UK; b Oxford Centre for Human Brain Activity, University of Oxford, Warneford Hospital, Oxford, UK; c Oxford Centre for Functional MRI of the Brain, University of Oxford, John Radcliffe Hospital, Oxford, UK

**Keywords:** Network, Dynamics, Magnetoencephalography, MEG, Sternberg task

## Abstract

The characterisation of dynamic electrophysiological brain networks, which form and dissolve in order to support ongoing cognitive function, is one of the most important goals in neuroscience. Here, we introduce a method for measuring such networks in the human brain using magnetoencephalography (MEG). Previous network analyses look for brain regions that share a common temporal profile of *activity*. Here distinctly, we exploit the high spatio-temporal resolution of MEG to measure the temporal evolution of connectivity between pairs of parcellated brain regions. We then use an ICA based procedure to identify networks of *connections* whose temporal dynamics covary. We validate our method using MEG data recorded during a finger movement task, identifying a transient network of connections linking somatosensory and primary motor regions, which modulates during the task. Next, we use our method to image the networks which support cognition during a Sternberg working memory task. We generate a novel neuroscientific picture of cognitive processing, showing the formation and dissolution of multiple networks which relate to semantic processing, pattern recognition and language as well as vision and movement. Our method tracks the dynamics of functional connectivity in the brain on a timescale commensurate to the task they are undertaking.

## Highlights

• A method is developed to track dynamic electrophysiological networks using MEG.
• Method based on ICA applied to timecourses measuring evolution of connectivity.
• Method allows a unique picture of transient networks that support cognition.
• Method validated in MEG data recorded during a Sternberg working memory task.
• Sensory networks observed include visual and sensorimotor.
• Cognitive networks relate to semantic processing, pattern recognition and language.

## 1. Introduction

Measurement of statistical interdependencies between neuroimaging signals has revealed a number of robust networks of functional connectivity in the brain (Beckmann et al., 2005, Corbetta, 1998, Fox and Raichle, 2007, Fox et al., 2005, Friston, 1994, Raichle et al., 2001, Smith et al., 2009). These networks, each with their own characteristic spatial signature, are thought to govern core mental processes with some supporting sensory integration and others associated with cognition or attention. Most networks are observed even in subjects at rest and are thus termed resting state networks (RSNs). Characterisation of RSNs is an emerging focus: not only does it offer new insight into how spatially separate regions integrate, RSNs (and functional connections in general) have been shown to be compromised in a variety of pathologies (Brookes et al., 2016, Friston, 1998, Guggisberg et al., 2008, Kessler et al., 2014, Palaniyappan and Liddle, 2012, Schnitzler and Gross, 2005, Stufflebeam et al., 2011, Tewarie et al., 2014, van Dellen et al., 2012) highlighting their clinical importance. To date, the majority of functional connectivity studies have been based on an assumption of stationarity; i.e. a connection between two regions is characterised by a single parameter derived over many minutes (or even hours) of data. However, the brain is a dynamic system and efficient function likely relies on the formation and dissolution of many hierarchical networks at rapid time scales, which support ongoing cognition. The characterisation of such transient networks represents a key goal in neuroscience. Increasing evidence (Allen et al., 2014, Baker et al., 2014, Baker et al., 2012, Chang and Glover, 2010, Chang et al., 2013, de Pasquale et al., 2010, de Pasquale et al., 2015, Hutchison et al., 2013, Karahanoğlu and Van De Ville, 2015, O’Neill et al., 2015b, Smith et al., 2012, Yaesoubi et al., 2015) suggests that such transient networks might be measured using neuroimaging. In this paper, we introduce a method which tracks the formation of functional electrophysiological networks, and use it to image the network formations associated with self-initiated movement, and working memory.

In order to track network dynamics effectively, we require a modality which can match the rapid timescales of the brain. Functional Magnetic Resonance Imaging (fMRI) has provided significant evidence of non-stationary connectivity (Hutchison et al., 2013), but its reliance on the blood oxygenation level dependant (BOLD) response means the fastest time scales are obfuscated by the latency and longevity of the haemodynamic response. In contrast, magnetoencephalography (MEG; Cohen, 1968, 1972) detects changes in extracranial magnetic fields induced by dendritic currents in the brain. Because signals are generated from electrical activity in neurons, it is possible to observe brain activity on a millisecond time-scale. This temporal richness has been exploited via the introduction of many techniques to quantify connectivity (Scholvinck et al., 2013) and correlation between the amplitude envelopes of band limited oscillations has shown that static networks, similar to RSNs observed using fMRI, can be observed (Baker et al., 2014, Baker et al., 2012, Brookes et al., 2011a, Brookes et al., 2011b, de Pasquale et al., 2010, Hipp et al., 2012, Hipp and Siegel, 2015, Liu et al., 2010, Luckhoo et al., 2012, O’Neill et al., 2015b, Wens et al., 2014b). More importantly, the temporal richness facilitates measurement of connectivity in short time windows, making MEG the method of choice for capturing transiently active networks; indeed exploitation of the spatiotemporal resolution of MEG has already shown that functional connectivity changes on the time scale of seconds (Brookes et al., 2014, O’Neill et al., 2015b) and even milliseconds (Baker et al., 2014). However, this area remains in its infancy; few methods are available and some remain limited by, for example, *a-priori* selection of brain regions or the significant problem of signal leakage between regions (Brookes et al., 2012b, Colclough et al., 2015, Wens et al., 2015). Further, few studies have attempted to probe the evolution of dynamic networks during a task.

In this paper, we undertake analysis of the timecourses of dynamic connectivity. Our method is based upon measurement of envelope correlation within small time-windows of data and between pairs of brain regions defined via cortical parcellation (Allen et al., 2014, Bola and Sabel, 2015, Colclough et al., 2015, Finn et al., 2015, Hassan et al., 2015, Hillebrand et al., 2012, Smith et al., 2015, Tewarie et al., 2014). By computing dynamic connectivity in a sliding window, we facilitate estimation of timecourses showing the evolution of functional connectivity at an intermediate timescale on the order of a few seconds. These connectivity timecourses are then analysed using independent component analysis (ICA). Past studies have shown ICA to be a valuable tool to elucidate networks: for example, timecourses of brain ‘activity’ can be acquired from multiple voxels and decomposed into a smaller number of temporally independent components, with a single component representing temporal signatures at multiple voxels; assessment of which voxels contribute to each component yields images of regions that share a temporal profile of *activity* (Brookes et al., 2011b). Here, distinct from this, having characterised the timecourses of connectivity, we decompose those timecourses in such a way that a single component represents an independent temporal signature shared by multiple connections. Assessment of the specific connections contributing to a component allows elucidation of networks of dynamically changing connectivities which share a single temporal signature. (I.e. we assume that multiple connections modulated in the same way in time are related functionally.) Our method characterises both the way in which connectivity evolves in time, and the spatial signatures of that evolution. In this way, we can uniquely track the dynamic behaviour of networks, on a timescale which is commensurate to the task they are undertaking.

## 2. Methods

### 2.1 Data Acquisition

Two separate MEG datasets were acquired. Both were approved by the University of Nottingham Medical School Research Ethics Committee.

• ***Dataset 1** – Self Paced Motor task:* 15 volunteers (9 male, aged 25 ± 4 years (mean ± SD)) were asked to execute a button press with the index finger of their non-dominant hand. Subjects were instructed to press the button infrequently; they were told that a button press should be executed approximately once every 30 seconds, but precise timekeeping was not important and they should not count the time between presses. A subset of these data has been used in prior publications (O’Neill et al., 2015b, Vidaurre et al., 2016).
• ***Dataset 2** – Sternberg task:* 19 healthy participants (10 male, aged 25±3 years) performed a Sternberg working memory task. Two *example* visual stimuli (abstract geometric shapes) were presented on a screen; each stimulus was shown for 0.6 s with 1 s between onsets. Following this, a period of 7 seconds was left, known as the *maintenance* phase, before a third (*probe*) stimulus was presented. If the probe stimulus matched either of the two example stimuli, the subject was told to execute a button press with their right index finger. Subjects received immediate feedback as to whether their response was correct. Trials were separated by 30 seconds of rest, where subjects fixated on a cross. 30 trials were presented to every subject.

MEG data were recorded using a 275-channel CTF MEG system (MISL; Coquitlam, BC, Canada) in synthetic 3^rd^ order synthetic gradiometer configuration; at a sampling rate of 600 Hz. Subjects were positioned supine. To ascertain the location of the head within the MEG helmet, three head position indicator (HPI) coils where attached to the subject at the nasion and preauricular points. These were energised periodically in order to track continuously the subjects head position. To allow coregistration of brain anatomy to the MEG sensor geometry, a measurement of the locations of the HPI coils relative to the scalp surface was created with a 3D digitiser (Polhemus; Colchester, VT). Anatomical images were acquired using either a 3T or 7T Philips Achieva MRI scanner (MPRAGE; 1 mm^3^ resolution). Coregistration of MEG data to anatomical MRI was then achieved by matching the digitised head surface to the equivalent surface extracted from the MRI.

### 2.2 Pre-processing and Source Reconstruction

**Fig. 1:**
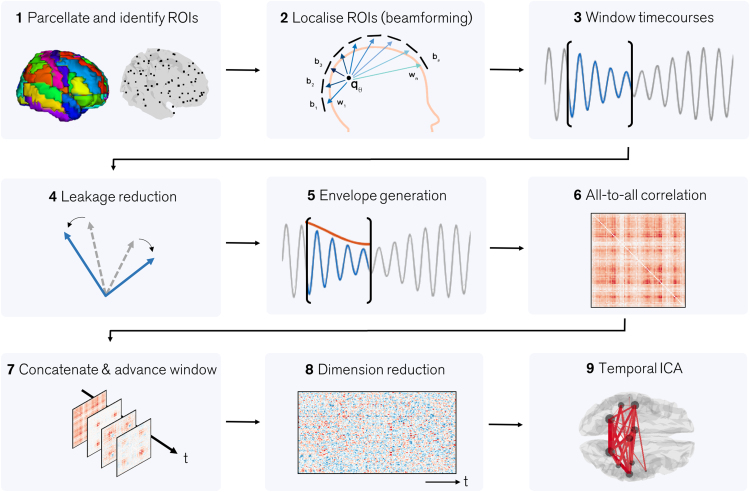
A schematic diagram describing the fundamental processing pipeline.

MEG data were initially inspected visually. Any trials deemed to contain excessive interference, for example generated by muscles or eye movement, were removed. In addition, datasets in which the subject's head moved more than 5mm (Euclidean distance) from its starting position were excluded. A schematic of the subsequent data processing pipeline is given in Fig. 1.

Following pre-processing, data were analysed using beamforming. The cortex was parcellated using the Automated Anatomical Labelling (AAL) atlas (Tzourio-Mazoyer et al., 2002) which had been modified by removing subcortical ROIs to leave 78 regions (Gong et al., 2009), and was transformed to each individual's brain geometry using FMRIB Linear Image Registration Tool (FLIRT) (Jenkinson et al., 2012). In order to obtain a representative time-series for every region, the centre of mass of each region was defined and used as a single representative location (Fig. 1 – step 1). MEG data were frequency filtered 1-150 Hz and source localised using an adaptive beamformer (Robinson and Vrba, 1998, Van Veen et al., 1997) in order to derive 78 source timecourses per subject, one for each AAL region (Fig. 1 – step 2). For beamforming, data covariance was defined in a frequency window spanning 1-150 Hz and a time window covering the entire experiment (Brookes et al., 2008). The covariance matrix was regularised using the Tikhonov method with the regularisation parameter set such that the regularised covariance matrix would have a condition number of 100. Forward fields were based upon dipole approximations (Sarvas, 1987) and a multiple local spheres head model (Huang et al., 1999). Dipole orientation was determined using a non-linear search for the optimal signal to noise ratio (SNR) (Robinson and Vrba, 1998, Sekihara et al., 2004). This process creates a source space data matrix, Q of dimension $n_{n}\times n_{s}$, where $n_{n}$ is the number of AAL regions (78) and $n_{s}$ is the number of samples.

### 2.3 Dynamic Functional Connectivity Analysis

We aimed to undertake a dynamic, all-to-all, functional connectivity analysis. This means that connectivity between all possible pairs of AAL regions is estimated, as a function of time, using a sliding window approach. Previous work (Baker et al., 2014, Hipp et al., 2012) has shown that functional connectivity is dependent on frequency band studied; for the self-paced motor study we employed a 13-30 Hz frequency window and for the Sternberg task, we employed a 4-30 Hz frequency window as it has been shown multiple frequency bands contribute to working memory (Brookes et al., 2012a). After frequency filtering, Q was segmented into overlapping time windows (Fig. 1 – step 3): we denote the data in a single window, $Q_{i}$, which has dimensions $n_{n}\;\times \;f\delta$. Here, i denotes window number, δ is the window width in seconds, and f is sampling frequency. In everything that follows δ = 6 s; the window was shifted in time by 0.5 s for each window number (i). In the self-paced motor task, time windows were centred between t = −12s and t = 12 s (where t represents window centre relative to the button press). There were 49 time windows per trial. In the Sternberg task, time windows were centred between t = −13s and t = 25 s (t represents window centre relative to trial onset). There were 75 time windows per trial. Within each window, we measured connectivity between all pairs of AAL regions.

In MEG, a significant confound for source space connectivity is that the ill-posed inverse problem, coupled with inaccuracies in the forward solution, cause a degree of spatial blurring and mislocalisation of sources. This means that two beamformer derived timecourses (e.g. from two regions) may exhibit significant correlation, purely due to ‘signal leakage’. Without careful control, this artifactually inflates estimated connectivity between regions (Maldjian et al., 2014). Signal leakage has been well studied, with a number of methods for leakage reduction now in place (Brookes et al., 2012b, Hipp et al., 2012, O’Neill et al., 2015a, Wens et al., 2015). Most methods rely on the fact that leakage manifests as a zero-time lag linear summation of underlying signals and for this reason orthogonalisation of beamformer projected signals (e.g. orthogonalisation of the rows of $Q_{i}$) results in the effective removal of leakage, albeit at the expense of genuine zero-lag connectivity. An elegant means to achieve orthogonalisation simultaneously over a set of multiple brain regions was recently proposed by Colclough et al. (2015). Here, signals from all $n_{n}$ regions are symmetrically orthogonalised within a single computation. The full mathematical details of this procedure can found elsewhere (Colclough et al., 2015). Briefly, the method involves two steps: First, a set of orthonormal time-courses, closest to the data matrix $Q_{i}$ and for which there is a simple analytic solution, is found. Second, the solution is finessed by iteratively adjusting the lengths and orientations of the corrected vectors until the solution is as close as possible to the uncorrected timecourses. The result is a set of matrices, $O_{i}$, whose rows contain the orthogonalised (windowed) time series for all 78 AAL regions (Fig. 1 – step 4). Note that the leakage reduction step was applied on each window separately (separate orthogonalisation for each i), rather than on the whole time series. This is because previous work (O’Neill et al., 2015b) has shown that leakage depends on signal to noise ratio, which changes in different time windows.

Following leakage correction, the amplitude envelopes of the windowed timecourses were found using Hilbert transformation. This resulted in a set of matrices $E_{i}$ whose rows contained the amplitude envelopes of orthogonalised neural oscillations (i.e. the envelope of the rows of $O_{i}$; Fig. 1 – step 5). Following this, Pearson correlation between amplitude envelopes was measured to form connectivity matrices, $R_{i}$, such that

1 $$R_{i}=\left[\begin{array}{ccc} r(e_{i1},e_{i1}) & \cdots & r(e_{i1},e_{in_{n}})\\ \vdots & \ddots & \vdots \\ r(e_{in_{n}},e_{i1}) & \cdots & r(e_{in_{n}},e_{in_{n}}) \end{array}\right],$$

where $e_{\mathrm{ik}}$ represents the vector of timecourse measurements in the *k*^th^ row of $E_{i}$ and r(x,y) represents the Pearson correlation coefficient between x and y. In other words, $R_{i}$ represents an $n_{n}\times n_{n}$ adjacency matrix representing connectivity between all AAL region pairs, in time window i (Fig. 1 – step 6). This process was repeated for all i, resulting in a set of N matrices per subject (one for each time window used; N represents the number of windows per subject). These matrices were concatenated both in time and over all subjects (Fig. 1 – step 7) to form an adjacency tensor, R, with dimensions $n_{n}\times n_{n}\;\times \;NN_{s}$, where $N_{s}$ represents the number of subjects.

### 2.4 Temporal ICA

The adjacency tensor, R, measures the temporal evolution of functional connectivity between all pairs of AAL brain regions. We now seek to apply ICA to derive independent temporal signatures of connectivity. To be able to perform ICA we need to reduce to the dimensionality of the data (Fig. 1 – step 8). We begin by vectorising each $n_{n}\times n_{n}$ matrix $R_{i}$ into a $1\times n_{n}^{2}$ row vector. Then, noting that the inherent diagonal symmetry in the adjacency matrix leads to redundancy, we remove that redundancy to generate the $1\times n_{c}$ vector $\rho_{i}$, where $n_{c}=\frac{n_{n}^{2}-n_{n}}{2}$ is the total number of unique connections modelled in $R_{i}$. These multiple row vectors are then concatenated in time to generate a new matrix Ρ such that $Ρ={\left[\rho_{1},\rho_{2},\ldots {,\;\rho }_{NN_{s}}\right]}^{T}$. This means that each column of Ρ represents the timecourse of an individual connection between 2 AAL regions. The dimensionality of this matrix was further reduced by prewhitening, and ICA was then used generate $n_{\mathrm{ic}}$ temporally independent components. Mathematically,

2 $${\hat{Ρ}}^{T}=\mathrm{AX},$$

where the rows of the $n_{\mathrm{ic}}\;\times \;NN_{s}$ matrix X represent temporally independent signatures of functional connectivity, collapsed across all connections. The mixing matrix, A, has dimension $n_{c}\times n_{\mathrm{ic}}$ and each column represents the contribution of each individual connection to the independent component. The ‘hat’ notation in Eq. 2 denotes that $\hat{Ρ}$ is an estimate of Ρ based upon the derived independent components. Here, Ρ was formed by concatenating all time windows, including all trials and subjects. The ICA decomposition (including prewhitening) was performed using the fastICA method (Hyvarinen, 1999) using a deflation approach with $n_{\mathrm{ic}}=10$. The spatial signature of each derived independent component was reconstructed based upon the columns of A (Fig. 1 – step 9).

### 2.5 Testing for task-modulated networks

The above analyses yield a set of $n_{\mathrm{ic}}\left(=10\right)$ networks, showing functional connections that share similar (independent) temporal profiles. The challenge now becomes to determine which of these represent genuine brain processes. The question of which independent components to keep and which reflect only noise is a problem in all ICA based methods. Here for simplicity, we sought to determine which components were modulated significantly by the tasks. Our procedure was based on previously described algorithms (Clare et al., 1999, Hunt et al., 2012, Winkler et al., 2014). We first defined a new matrix, $\bar{X}$, containing $n_{\mathrm{ic}}$ trial averaged independent component timecourses (i.e. $\bar{X}$ is just X averaged over all trials in all subjects; i.e. the ‘bar’ notation represents a trial average.) The size of $\bar{X}$ was $N_{\mathrm{Trial}}\times n_{c}$ (where $N_{\mathrm{Trial}}$ represents the number of time windows per trial; 49 for the self-paced task and 75 for the Sternberg task). Following this, we constructed two empirical null distributions:

1. In the first case, a ‘sham’ matrix, ${\tilde{X}}_{\mathrm{flip}}$, was generated in exactly the same way as $\overset{̅}{X}$, but prior to averaging over trials, half of the subjects were selected randomly and the sign of their contribution to the independent components in X was ‘flipped’ (i.e. multiplied by −1). We reasoned that, if the independent components were not related to the tasks, this sign flipping would have no effect on the magnitude of the trial averaged timecourses, and therefore the magnitudes of fluctuations in ${\tilde{X}}_{\mathrm{flip}}$ and $\bar{X}$ would match. However, if the independent components contained trial-onset-locked increases or decreases in connectivity, which were robust across subjects, then these would be maintained in $\bar{X}$ but diminished in ${\tilde{X}}_{\mathrm{flip}}$. This procedure was repeated 6435 times for the self-paced data (reflecting $C_{7}^{15}$ where $C_{k}^{n}=\left(n!\right)/\left(k!\left(n-k\right)!)\right)$ and 92,378 times for the Sternberg data $\left(C_{9}^{19}\right)$, giving all possible realisations of ${\tilde{X}}_{\mathrm{flip}}$, with a different set of subjects selected each time. In this way an empirical null distribution was constructed against which the magnitude of signal fluctuations in $\bar{X}$ was tested. Note that this ‘sign-flipping’ permutation approach has been employed in previously published work (Hunt et al., 2012, Winkler et al., 2014).
2. In the second case, we reasoned that if no task induced response was expected, then the trial onset times would be meaningless. The trial averaging procedure was again repeated, however rather than the trial averaged data ($\overset{̅}{X}$) defined based on genuine trial onsets, a ‘sham’ averaged dataset, ${\tilde{X}}_{\mathrm{onset}}$, was defined based on randomly selected ‘sham trial onsets’. 6000 realisations of ${\tilde{X}}_{\mathrm{onset}}$ were created, again allowing for the generation of a null distribution for each time point and each independent component.

An independent component was deemed significant if, at any one time point in the trial average, the associated column of $\overset{̅}{X}$ fluctuated such that it fell outside a threshold defined by the null distributions for both tests (sign-flip and randomised onset). The threshold for significance was defined at 0.05, however this was corrected in three ways: First a 2-tailed distribution was allowed, meaning that fluctuations in the columns of $\overset{̅}{X}$ could be both greater than, or less than the null distributions. Second, we Bonferroni corrected for multiple comparisons across the 10 independent components (for both tasks). Third, we Bonferroni corrected across independent temporal degrees of freedom. Note that the 6 s sliding window used to estimate connectivity means that the number of temporal degrees of freedom in the averaged trial is substantially less than the number of time points. Here, we assumed that a single temporal degree of freedom was added each time the window shifts by more than half of its width (i.e. when adjacent windows share less than 50% overlap). This meant a total of 8 temporal degrees of freedom in the self-paced data and 12 in the Sternberg data. Thresholds were therefore set at (0.05/(2×10×8) = 0.0003) for the self-paced experiment and (0.05/(2×10×12) = 0.0002) for Sternberg experiment. [It is important to note that these Bonferroni corrections are rigorous, but nevertheless are likely conservative; in future uses of this technique methods based on, for example, maximal statistics (Sekihara et al., 2005) might offer a less conservative approach.]

## 3. Results

In what follows, we demonstrate the utility of our method in real MEG data, however our methodology was also tested in simulation. These results can be found in the Appendix and supplementary material.

**Fig. 2:**
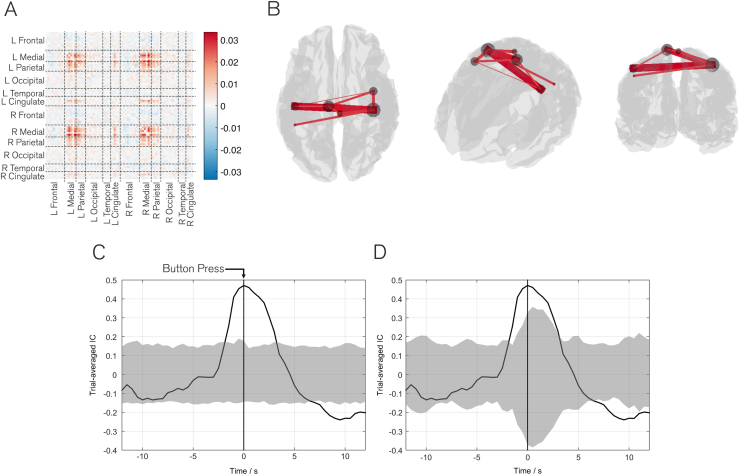
Results of the self-paced experiment. A) Matrix representation of the network; the ordering of the 78 AAL regions is overlaid. Note that the values in the matrix are the ICA derived mixing coefficients. B) 3D representation of the same network, thresholded for visualisation. Lines show connections, with thicker lines indicating stronger connections. Circles represent the summed magnitude of connectivity between that region and the rest of the brain. C) Time evolution of the network during the self-paced finger movement task, averaged across trials in all subjects (black line). Time represents the position of the centre of the 6 s window, relative to the button press at t = 0 s. The grey shaded region represents the null distribution based on a hypothesis that the response is not time locked to the button press. D) Sign-flip analysis, again showing the mean response across all 15 subjects (black line). The grey shaded area represents the null distribution based on a null hypothesis that the modulation is driven by a small number of subjects. Significance (p_corrected_<0.05) is attributed if the black line appears outside the null distribution in both C and D. Note that the network clearly represents the primary somatosensory, motor and supplementary motor regions and demonstrates significant modulation with the task. (An interactive version of this Figure can be found at http://nottingham.ac.uk/~ppzgo/ica_nets.).

Fig. 2 shows the results of our method applied to the self-paced data. Although 10 independent components were derived, here we present one of two networks that demonstrated significant task modulation as an example. The other 9 networks are shown in supplementary material. Fig. 2A shows a matrix representation of the network. The ordering of the 78 AAL regions is overlaid for reference. Fig. 2B shows the same network represented in 3D and thresholded (70% of the maximum connection strength) for clarity. Both the matrix and 3D visualisation show clearly that the network is centred on the right primary somatosensory cortex and highlights strong connections both between sensory and motor areas, the supplementary motor regions and left primary sensorimotor cortices. Fig. 2C shows the time evolution of this network, represented as the corresponding trial averaged independent component in ${\overset{̅}{X}}_{j}$. Time values on the x-axis represent the centre of the window with respect to the button press, which was at time zero and is shown by the vertical line. The grey area represents the null distribution generated by randomising the trial start times (${\tilde{X}}_{\mathrm{onset}}$). Significant modulation of connectivity occurs during the task; although this begins ~3 s prior to the button press, recall that the window size used was 6 s, generating inherent temporal uncertainty. Fig. 2D mirrors the results in Fig. 2C, but the grey area shows the empirical null distribution derived using the ‘sign-flip’ analysis (${\tilde{X}}_{\mathrm{flip}}$). Again, the black line represents the average response across all subjects, and the grey distribution is the 95^th^ percentile threshold for the null distribution. Overall, it is clear that a network, representing primary somatosensory and motor regions, is modulated significantly by the task. Given that the task requires both movement, and elicits a tactile response (since the subject will feel the button press), this network is plausible. A second component, representing the visual network, also showed significant modulations around the time of the button press, this can be found in the supplementary material.

**Fig. 3:**
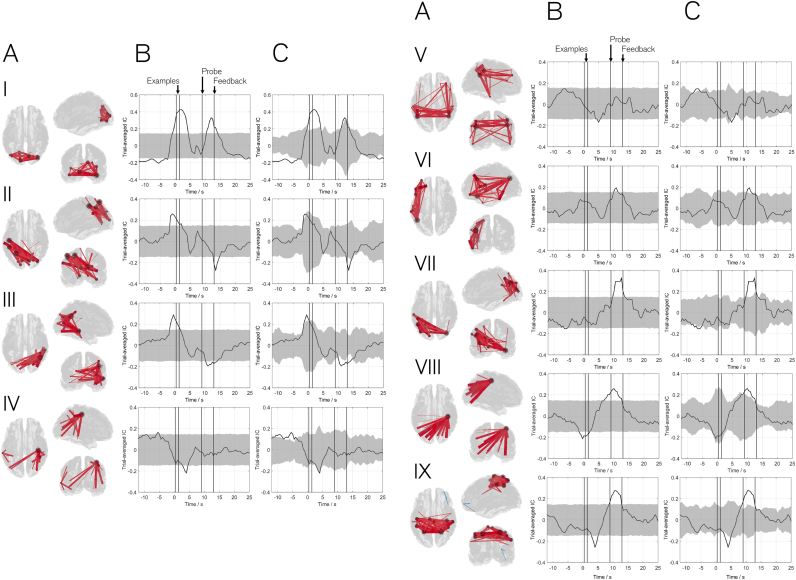
Results of the Sternberg experiment. The separate columns show A) 3D network visualisation. B) The average timecourse across 19 subjects with null distribution based on randomised trial start times. C) Equivalent to B but null distribution based upon sign flipping. Rows I to IX show the 9 networks which modulate significantly with the task, including I) primary visual; II) Visual to left tempero-parietal; III) Visual to right tempero-parietal; IV) Visuomotor V) Somantic; VI) Language; VII) Refined Visual to left tempero-parietal; VIII) Refined visuomotor; IX) Sensorimotor. Note how the timings allow a temporal sequence of network involvement to be deduced. (An interactive version of this Figure can be found at http://nottingham.ac.uk/~ppzgo/ica_nets.).

Fig. 3 shows the results of our method applied to the Sternberg dataset. Clearly, the increased cognitive load evoked by the Sternberg tasks elicits changes in a greater number of brain networks, and this is shown by 9 of the 10 networks derived demonstrating significant task induced modulation. Fig. 3 is laid out such that the columns represent: (A) a 3D network visualisation, (B) the average timecourse (19 subjects) alongside a null distribution based upon ${\tilde{X}}_{\mathrm{onset}}$ and (C) the average timecourse alongside a null distribution based upon ${\tilde{X}}_{\mathrm{flip}}$. The separate rows (I through IX) show the 9 networks which modulate significantly.

Unsurprisingly given the visual nature of the task, the four networks showing early task modulation all involve the visual areas. These are shown in rows I to IV of Fig. 3. Specifically, row I depicts a primary visual network whose connectivity increases during presentation of the two example stimuli (and also during the probe). Rows II and III show left and right lateralised connections between the primary visual areas and tempero-parietal regions, with both networks exhibiting an early increase in connectivity peaking immediately before presentation of the example stimuli. Row IV shows a visual to right motor cortex connection, which demonstrates a significant drop in connectivity during presentation of the example stimuli. Transient networks forming in later task phases are shown in rows V to IX. Row V shows a breakdown in connectivity during the task maintenance phase within a bilateral parietal, temporal and frontal network. Interestingly, this network captures some areas associated with the default mode network whose activity is known to decrease with a cognitive task. However, the network also captures areas associated with semantic processing and is thus termed the semantic network. Row VI highlights a left lateralised network that incorporates regions of temporal, parietal and frontal cortex. The regions implicated are strongly associated with the production of language as well as shape and pattern recognition; this is consistent with peaks in connection strength occurring during presentation of the stimuli. Row VII shows a refined visual to temporal and parietal network, similar to that in III but this time peaking around the time of the probe stimulus. Row VIII again shows a visual to motor connection (similar to IV), and finally row IX shows the sensorimotor network which becomes most strongly connected around the time of the button press response (in agreement with our result in Fig. 2). It is noteworthy that the brain regions implicated in these networks incorporate the primary sensory cortices, association areas, and cognitive networks that would be associated with semantic processing, pattern recognition and verbalisation, and so these networks are plausible given the task. This is addressed further in our discussion.

## 4. Discussion

This paper has introduced a novel ICA based method which, when applied to MEG data, allows characterisation of transiently forming and dissolving electrophysiological networks in the brain, at time-scales much faster than could be achieved using fMRI. Previous MEG-ICA-network approaches typically look for brain regions whose activity, measured as a function of time, covaries. Here distinct from this, we measure the temporal evolution of functional connectivity between regions and use temporal ICA to cluster together *connections* that share similar temporal profiles. In this way, we identify networks of connections whose temporal dynamics covary, with no prior assumptions. This allows us to identify where and when significant modulations in connectivity occur, We have verified our method in simulation (see appendix and supplementary material) and using a simple finger movement task. Moreover, we have shown that our method allows generation of a unique picture of cognitive processing, showing the formation and dissolution of multiple brain networks required to allow subjects to complete a working memory task.

The results generated by our method are of significant neuroscientific interest and warrant further discussion. However prior to this, two key points regarding the method should be understood: Firstly, the timecourses shown in Fig. 2, Fig. 3 depict increases and decreases in *connectivity*. In other words, the peaks refer to points in time when two or more regions defining a network are most correlated. Just because regions are not correlated at some particular point in time, does not necessarily mean that those regions are not engaged by the task. This is an important point since many of the regions implicated by our networks are likely to be engaged constantly throughout the Sternberg task, but may only connect to wider networks at specific points in time. Second, recall that there is inherent temporal smoothness in the method. Despite the excellent temporal resolution of MEG, a reasonable data window is required in order to derive reliably each individual adjacency matrix $R_{i}$ (see also below). Here we employ a 6 s window width, meaning that features in a timecourse have an inherent temporal uncertainty of ±3 s. This means that, for example in the self-paced motor task where connectivity appears to increase before the button press, there is a degree of ambiguity; this could be representative of preparatory effects, or could result simply from the limited temporal resolution of the method. This temporal resolution is lower than other MEG based connectivity techniques, for example the Hidden Markov model introduced by Baker et al. (2014). However this ±3 s resolution allows us to investigate the functional connections which evolve at an intermediate scale between the infra-slow connection evolution and the millisecond scale; this scale remains significantly higher than would be possible using techniques such as fMRI where a 6 s window would not facilitate sufficient data capture to accurately define connectivity. With these two considerations in mind it proves instructive to discuss the primary results of our method applied to the two datasets used. Fig. 2 shows clearly that a network of brain connections involving primary somatosensory and motor cortices, as well as supplementary motor areas, can be identified based upon our self-paced finger movement task. Furthermore, this network of connections modulates significantly with the button press. Although simple, this result confirms the validity of our method by depicting clearly the primary sensorimotor and motor planning regions. The fact that only one other network (visual) modulates significantly with the task also helps to verify that the statistical method used is capable of rejecting those networks that do not show task modulation.

In the Sternberg task, the formation of networks encompassing visual (Fig. 3,I) and sensorimotor (IX) regions is consistent with the presentation of visual stimuli and execution of the motor response (Metzak et al., 2011, Woodward et al., 2013, Yamashita et al., 2015). Nodes in the occipital lobe typically include a lateral component which supports the notion that lateral occipital cortex (LOC) is specialised for object shape recognition (Corbetta et al., 1991, Grill-Spector et al., 2001, Haxby et al., 1994, Kourtzi and Kanwisher, 2001). Other networks encompass areas thought to be responsible for the higher level cognition required for successful completion of the Sternberg task. The Angular Gyrus (AG) is particularly evident in the majority of these networks. Structurally this region has been identified as a centrally connected hub serving multiple sub-networks. This hub has also been identified functionally in a variety of task-positive contexts ranging from semantic processing to numerical calculation. A unified account of AG function is presented by Seghier (2013) who suggests that the AG is an integration site receiving input from sensory (Demonet et al., 1992, Vandenberghe et al., 1996), memorial (Geschwind, 1965) and higher-level nodes. We speculate that the extent of our higher order networks is in agreement with this model of AG function. Notably, the dorso-lateral pre-frontal cortex (DLPFC) is recruited in network V, connecting bilaterally with the AG. The left and right DLPFC are well established in the literature as controlling executive-attention function in working memory (Barbey et al., 2013, Kane and Engle, 2002), with the right DLPFC being shown to be sensitive to shape in particular (Nystrom et al., 2000). This network also incorporates bilateral inferior temporal gyri, regions considered important for semantic processing (Vigneau et al., 2006). This leads us to name this network as a ‘semantic network’. This network was also seen in a recent study by Shine et al. (2015), who saw the connectivity between the DLPFC and ventral visual regions vary with cognitive load in a working memory task. A second cognitive network (VI) has been termed a ‘language network’. Although stimuli were abstract shapes, participant feedback suggests a ‘naming’ strategy was used in the majority of cases. If a verbalization strategy was employed by the participants to aid in memory encoding, then nodes of the language network may be implicated. Indeed, this left lateralised network is anchored in the AG with extensions to the inferior frontal gyrus (IFG), inferior temporal gyrus and a number of nodes spanning the inferior to superior precentral gyrus. These regions are consistent with previous accounts of semantic cognition (Binder et al., 1997, Demb et al., 1995, Derrfuss et al., 2004, Kang et al., 1999, Vigneau et al., 2006). Furthermore, this effect was also seen by Caminiti et al. (2015) in a similar (abstract shape based) working memory task. These authors also considered a verbalisation strategy as the likely interpretation. Finally, two networks (IV & VIII) show ipsilateral motor connectivity with an extended network of occipital and parietal nodes. This is unusual considering the expected motor response would be in the contralateral hemisphere. However, the 4Hz-30Hz frequency band used encompassed alpha and beta oscillations and it is possible that, to suppress ipsilateral motor activity, alpha oscillations are increased (Brinkman et al., 2014). Overall, the transient networks induced by the Sternberg task are plausible given the previous literature on working memory and sensory processes.

The applications of these methods in the clinical domain are promising. It is well known that neural oscillations, upon which these connectivity metrics are based, are perturbed in a wide variety of developmental, psychotic and degenerative disorders. Similarly the efficacy of aspects of cognition such as working memory are also significantly reduced in many patients. It follows that the time-evolving networks of functional connectivity derived in the present paper may differ between control and patient groups, and such findings might offer a novel means to understand the neural substrates underlying cognitive decline in disease. Future studies will therefore likely be able to employ the methodology presented here to highlight dysconnectivity in disorders such as schizophrenia, where abnormal recruitment of brain regions might be expected.

### Methodological considerations

Our algorithm allows detection and characterisation of transiently forming task induced electrophysiological networks. In achieving this, two core parameters require setting, the window width (here 6 s) and the number of independent components (here 10). Both warrant further discussion. A judicious selection of window width is important, and represents a trade-off between temporal resolution and the accuracy of the derived adjacency matrices. Here, separate elements of the adjacency matrices are based upon temporal correlation of envelope signals within the window. It is well known that the accuracy of correlation between two variables (*r*) relates to the number of degrees of freedom (η) in the underlying data; specifically if one assumes no underlying genuine correlation between two timecourses then standard deviation of correlation, $\sigma \left(r\right)=1/\sqrt{\eta }$; i.e. the variability (noise) inherent in the adjacency tensor is increased as η is decreased. Further, the number of degrees of freedom in a windowed envelope timecourse is unrelated to the number of sample points (or sampling frequency). In fact, Fourier theory shows that for envelope data, an upper limit on degrees of freedom is given by $\eta =B_{w}\delta$, where δ is the window width and $B_{w}$ represents bandwidth of the carrier signal (i.e. for a 13-30 Hz beta envelope, $B_{w}\;=\;17\mathrm{Hz}$). This means that $\sigma \left(r\right)=1/\sqrt{B_{w}\delta }$; in other words, adjacency matrix noise is increased by either reducing bandwidth of the carrier signal, or the window width. Typically, bandwidth is set by the scientific question to be asked (e.g. one might be interested in beta band networks, such as in the self-paced motor study), and therefore δ must be set to reduce the random noise to an acceptable level. Here σ(r)=0.1 for the self-paced data and σ(r)=0.08 for the Sternberg data, which was deemed acceptable. Future studies should bear this calculation in mind. The selection of the number of independent components is less well prescribed; this is not a limitation of our algorithm directly, but rather is a fundamental question for all ICA methodologies. In the present work we selected 10 components based on our previous experience, although varying this parameter in our current work made little difference to the overall results. Further, here we select which independent components to keep based upon those networks which modulate significantly with the task. However, just because a network does not modulate with the task does not necessarily mean that this network is not genuinely representative of connectivity. Future work should therefore seek other methods to determine validity of networks, particularly if the present algorithm was to be used for resting state investigation.

In addition to parameter selection, there are three other core components of the method that warrant discussion; namely, the choice of cortical parcellation, the underlying source space projection method, and the choice of connectivity metric. First, regarding the AAL parcellation, this was chosen based on its successful use in previous MEG investigations (e.g. Tewarie et al., 2016). However, our method could be used with any cortical parcellation, provided that the number of regions is sufficiently low, and those regions are sufficiently well separated to ensure that the windowed data matrices, $Q_{i}$, are of full rank. (This is a requirement of the orthogonalisation procedure (Colclough et al., 2015).) It is noteworthy that the separate AAL regions vary markedly in size; our use of a single point location, based on the centre of mass of the region, may therefore mean that some regions are better represented than others. This suggests that brain regions that are poorly represented may be missing from the networks shown. For example, one would expect that areas in the ventral visual pathway (e.g., fusiform gyrus) to involved in our Sternberg task. However, they were not core to any of the networks shown. A likely reason is that they are missed by the cortical parcellation and single point (centre of mass) representation. The future use of brain parcellations based on functional MRI (Craddock et al., 2012), MEG, multmodal (Glasser et al., 2016), or even a-priori (literature based) knowledge of brain regions involved in a task may therefore prove instructive. Secondly, for source localisation, we chose to employ a beamformer spatial filtering procedure. Beamforming is a popular method of inverse solution and has been shown previously to be particularly useful in the characterisation of neural oscillations. Further, beamforming has been used successfully in the measurement of static (Brookes et al., 2011a) and dynamic (Baker et al., 2014) functional connectivity. The reasons for the success of this algorithm in such studies has been addressed at length in previous papers, and will not be repreated here. However, we point out that other inverse solutions could be substituted for beamforming in the present processing pipeline, and would likely generate similar results. Thirdly, we chose an envelope correlation procedure as our estimator of functional connectivity between regions. This procedure has been successful in elucidating electrophysiological networks of functional connectivity (Colclough et al., 2016), particularly in the study of the electrophysiological basis of haemodynamic networks (Tewarie et al., 2016). However, other methods (for example those based on fixed phase measurements between regions) are available; these should not be considered competitor techniques but rather ways to probe a different type of functional connectivity (Scholvinck et al., 2013). For example, using a time-varying multivariate autoregressive model, it has been demonstrated that task-dependent brain states can be identified in a finger tapping task, and correspond to unique cross-spectral (i.e. coherence) patterns (Vidaurre et al., 2016). Although at present this method this is limited (computationally) to pairs of brain areas, whereas our method in this paper is whole-brain. The two methods may be combined in the future. Indeed, the adjacency matrices derived in our methodology could easily be substituted for similar adjacency matrices derived using any alternative metric (assuming sufficiently high signal to noise ratio), and transient networks probed.

We note that there is significant variability in the timecourse of connectivity across subjects. This is demonstrated in null distributions formed based upon randomized sign flip in half of the subjects; i.e. if connectivity change was equal in all subjects, the sign flip would perfectly cancel any task response, and no variation over time would be seen in the in null distributions (grey shaded regions in Figs. 2D and 3C). The fact that null distributions follow, to a degree, the genuine timecourses shows, in the Sternberg and self-paced experiments, a marked variation in connectivity profile over subjects. In fact, relatively poor within and between subject reliability of (static) MEG connectivity measurements has been shown previously. For example, Wens et al. (2014a) show that whilst group level static connectivity within several well-known distributed networks is stable, there is significant variability at the individual subject level. Similarly Colclough et al. (2016) tested the cross session repeatability of a large number of static functional connectivity measurements, showing clearly that although group level inference is reliable, network metrics can be very variable across individuals. In addition, Tewarie et al. (2016) used MEG networks to predict those observed in fMRI; whilst predictions were robust at the group level, they fared less well within individuals. Interestingly, these variations across subjects may not be due to stochastic noise, but rather identifiable intrinsic processes which are subject specific (Finn et al., 2015). Given these previous findings of large inter-individual differences in static connectivity, it is not surprising that dynamic functional connectivity metrics presented here also exhibit relatively high inter-individual differences. There are a number of possible explanations for this. Firstly, our measurement of connectivity itself (i.e. the dynamic adjacency matrices) are based only on 6 s of unaveraged MEG data. Given the relatively low SNR of MEG data it is possible that reliability is only realised with large quantities of data – hence the requirement for large subject cohorts. Second, source localisation could affect the robustness of connectivity; here we use beamforming alongside the AAL atlas, a technique well established by previously published work. However, a limitation is that if a specific region, e.g. left motor cortex, is mislocalised (e.g. due to a poor forward model in one subject) then the signal derived would no longer be representative of that region. This potential confound would add markedly to variability over subjects. Thirdly, the reliability of the amplitude envelope correlation metric itself could be questioned. However, Colclough et al. (2016) showed that of all of the MEG based connectivity metrics, AEC fared well in terms of robustness over repeated measures. Finally, this variability could genuinely reflect the variability across individual subjects in terms of the neural network mechanisms used to carry out the tasks undertaken. Ultimately, if techniques like the one presented here are to be useful clinically, then we must derive means to ensure their robustness in individuals. Further effort is thus needed in this area.

## 5. Conclusion

The characterisation of dynamic electrophysiological brain networks, which form and dissolve in order to support ongoing cognitive function, is one of the most important challenges in neuroscience. Here, we introduce an ICA based method for measuring such networks in the human brain using MEG. Previous MEG-ICA network analyses look for brain regions that share a common temporal profile of activity. Here distinctly, we measure the temporal evolution of connectivity between region pairs and use ICA to identify clusters of *connections* that share an independent temporal profile. The validity of our method was demonstrated in simulation and in a self-paced finger movement paradigm, showing that a sensorimotor network can be distinguished. The broader applicability of our method was demonstrated by its application to a Sternberg task. We have shown that our method allows generation of a unique picture of cognitive processing, showing the formation and dissolution of the brain networks required to allow subjects to complete the task. This represents a significant step forward in the characterisation of brain network connectivity and will prove to be a key tool in the future investigation of healthy brain networks, and their breakdown in a variety of pathological conditions.

## Figures and Tables

**Fig. A1:**
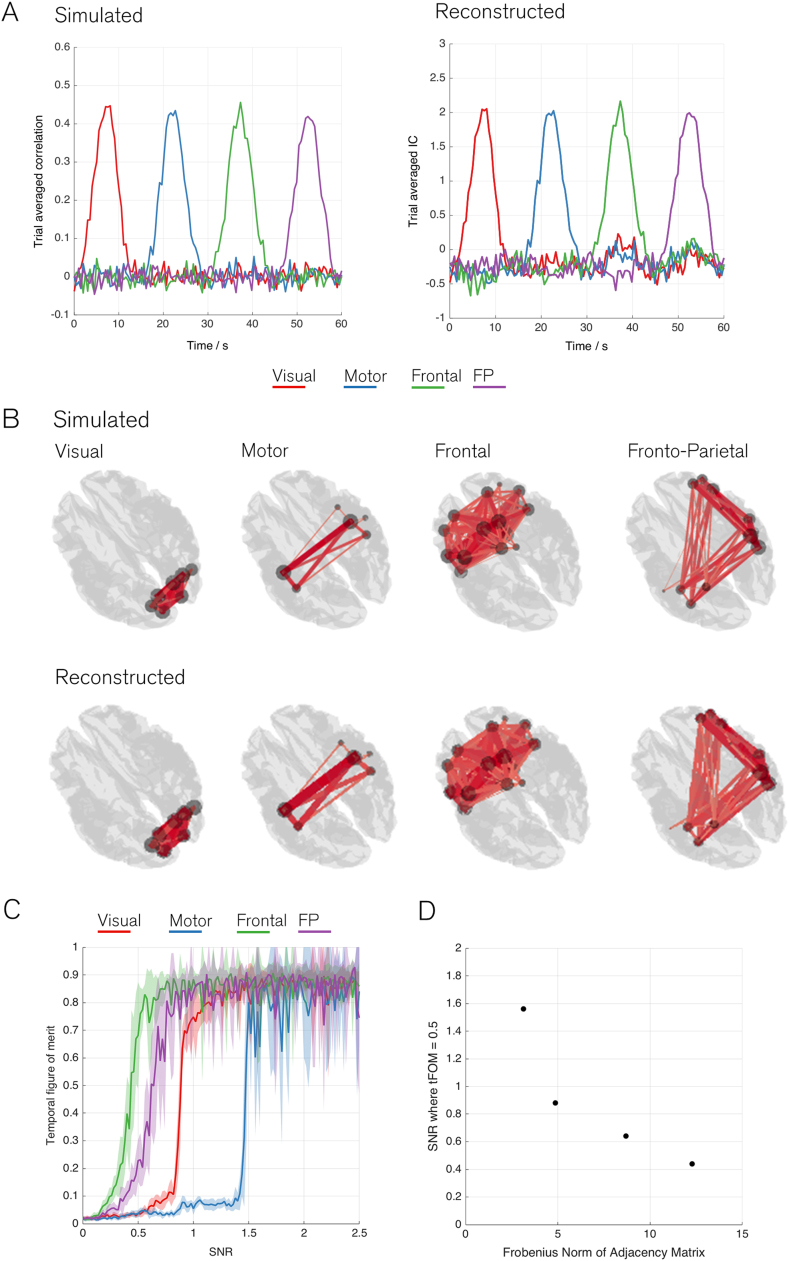
Simulation results. A) The trial averaged simulated timecourses (left) and the trial averaged independent components (right) (SNR = 3). B) 3D visualisations of the simulated (top) and ICA reconstructed (bottom) network topographies (top row). C) Temporal figure of merit scores plotted as a function of SNR for all 4 simulated networks. Note that accurate reconstructions are observed whilst SNR is above a critical threshold level; threshold differs for each network. D) The relation between SNR threshold (defined as when the figure of merit falls below 0.5) and Frobenius norm of the network adjacency matrix. Note that networks with a larger number of connections fare better in low SNR conditions.

## Appendix Validation by simulation

The validation of our methodology provided in this paper is centred upon its application to real MEG data. However, it also proves instructive to examine the performance of our methodology in simulation. The performance of envelope correlation as a means to examine connectivity in short time windows has been addressed extensively in previous work (Brookes et al., 2014, O’Neill et al., 2015b) and will not be repeated here. However, the ability of ICA, applied to timecourses of connectivity, to extract the spatiotemporal signatures of multiple brain networks has not yet been validated. In what follows, we test the extent to which ICA characterises a set of simulated networks which are obfuscated by noise. Here, key findings are presented; a full description of the methods and extended results can be found in supplementary material.

### Simulation Methods

We simulated an adjacency tensor $R_{\mathrm{sim}}$ based on a weighted combination of “true” connectivity ($S_{\mathrm{sim}}$) and interference ($I_{\mathrm{sim}}$). To generate $S_{\mathrm{sim}}$, four spatially distinct networks were constructed based on a previous study (Brookes et al., 2015). The spatial patterns of connectivity, reflecting visual, sensorimotor, superior frontal and fronto-parietal networks, were each represented by an adjacency matrix; 3D visualisations are shown in Fig. A1B. A timecourse of connectivity for each network was also generated; these comprised 60 minutes of periodically occurring Hanning windows. Amplitudes were set to 0.45, the full width half maximum was 6 seconds, and onsets were set to the 3rd, 18th, 33rd and 48th second of every minute. A single trial was defined as 1 minute in length. The trial averaged simulated data are shown in Fig. A1A (left hand panel). The timecourses were multiplied by their respective network adjacency matrices to create $S_{\mathrm{sim}}$. The interference tensor, $I_{\mathrm{sim}}$ was generated by source reconstructing 60 minutes of recorded empty room MEG data onto a simulated brain geometry, and measuring amplitude envelope connectivity as described in our methods section. This generated structured (but spurious) networks of inference. Both $S_{\mathrm{sim}}$ and $S_{\mathrm{sim}}$ were sampled at 2 Hz to reflect the analysis pipeline described above. The two tensors were combined thus,

A1 $$R_{\mathrm{sim}}=\frac{S_{\mathrm{sim}}}{1+(\frac{1}{\mathrm{SNR}})}+\frac{I_{\mathrm{sim}}}{1+\mathrm{SNR}},$$

where SNR represents the effective signal to interference ratio of the simulated data (see supplementary material for details).

In order to test the effectiveness of ICA at extracting the network time courses in the presence of interference, the SNR parameter was allowed to vary and a temporal figure of merit derived to characterise how well a single component represents a simulated time course. This was defined as the correlation between a simulated network and its best matching independent component, minus the mean correlations between the reconstructed timecourse and all other simulated components. This value ranges between 0 and 1. Note that the figure of merit penalises both poor representation and degeneracy, i.e. a low value is computed if a single component represents multiple simulated networks.

### Simulation Results

Fig. A1A shows the trial averaged independent components from of 4 out of 7 reconstructed components. The associated spatial patterns are shown in Fig. A1B. Note strong temporal and spatial resemblance to the simulated connectivity which is also reflected by figure of merit scores of 0.84, 0.91, 0.84 and 0.94 for the 4 simulated networks. Fig. A1C shows the results of varying the SNR of the simulated data between 0 and 2.5 in steps of 0.02. 10 simulations were run for each SNR value. As would be expected, at high SNR the figure of merit scores are high, meaning that our simulated networks are reconstructed faithfully. However, a sharp transition below a minimum ‘threshold’ SNR is observed below which simulated networks are irretrievable. Interestingly, a different threshold value can be observed clearly for each network, and this relates to the number of connections which exist in a network; the more connections present, the lower the SNR that can be tolerated. This is reflected in Fig. A1D, where the threshold (defined as the SNR which generates a figure of merit score of 0.5) is plotted against the Frobenius norm of the network adjacency matrices (which here indirectly represents number of connections). Fig. A1D shows clearly that SNR is a monotonically decreasing function of norm, making the important point that networks with large spatial extent (lots of connections) can be reconstructed using lower SNR data. In other words, our method will preferentially reconstruct networks with larger spatial extent; future applications of this method should bear this potential confound in mind.
