# Supplementary material for: Measurement of dynamic task related functional networks using MEG
Source: Neuroimage. 2017 Feb 1;146:667–78. doi: 10.1016/j.neuroimage.2016.08.061 (PMC5312793; doi:10.1016/j.neuroimage.2016.08.061)
Supplement: Supplementary file 1 — Supplementary material [file mmc1.pdf]

Measurement of Dynamic Task Related Functional Networks using MEG

George C. O'Neill, Prejaas K. Tewarie, Giles L. Colclough, Lauren E. Gascoyne, Benjamin A.E. Hunt, Peter G. Morris, Mark W. Woolrich and Matthew J. Brookes

Supplementary Material: Validation via Simulation

Overview:

The validation of our methodology provided in the main manuscript is centred upon its application to real MEG data. However, it also proves instructive to examine the performance of our methodology in simulation. The performance of envelope correlation as a means to examine connectivity in short time windows has been addressed extensively in previous work (Brookes et al, 2014; O'Neill et al 2015) and will not be repeated here. However, the ability of ICA, applied to timecourses of connectivity, to uniquely extract the spatiotemporal signatures of multiple brain networks has not been validated. In what follows, we test the extent to which ICA successfully characterises a set of simulated networks which are obfuscated by realistic noise. In addition to a simple simulation validating our method, we also probe the effect of poor signal to noise ratio and shared signal variance across networks. We will show that our methodology can indeed reconstruct accurately both the spatial and temporal network profiles. However, we also show that accurate reconstruction depends critically on signal to noise ratio, the number of connections in a network, and (intuitively) the extent to which underlying network timecourses are independent.

Simulation Methodology

We aimed to simulate data at the level of the adjacency tensor, $\boldsymbol{R}$. Recall that this tensor is representative of a set of adjacency matrices, measured at different points in time and characterising moment to moment changes in connectivity between parcellated regions. The simulated tensor, $\boldsymbol{R_{sim}}$, was constructed based upon 2 separate tensors, one representing simulated networks $\boldsymbol{S_{sim}}$, and the second representing interference $\boldsymbol{I_{sim}}$.

To generate $\boldsymbol{S_{sim}}$, four spatially distinct networks were constructed based on a previous MEG study (Brookes et al., 2015). The spatial patterns of connectivity were each represented by an ($n_n \times n_n$) adjacency matrix, $\boldsymbol{P}_{\mathrm{j}}$, (where $j = 1-4$ and n_n is the number of AAL regions). These matrices, alongside a 3D visualisation of the network, are shown in columns 1-4 of Figure S1 and reflect visual, sensorimotor, superior frontal and fronto-parietal networks. In addition to the spatial signature, we simulated the time evolution of dynamic connectivity in each network. (i.e. we allow each of these networks to form and dissolve on a timescale commensurate to brain activity). In order to do this, Each network was assigned a simulated timecourse $m_j(t)$ which was given by

$$m_j(t) = \alpha f_{1j}(t) + \beta f_{2j}(t), \qquad \text{[S1]}$$

Here, $f_{1j}(t)$ is the modulation function for the jth network, which was represented by a Hanning window of unit amplitude, periodically occurring once every minute for 60 minutes. The full width half maximum (FWHM) of the Hanning window was 6 seconds. For the visual, sensorimotor, frontal and fronto-parietal networks, the onset of the Hanning window was on the 3rd, 18th, 33rd and 48th second of every minute respectively. $f_{2j}(t)$ represents uncorrelated Gaussian noise which was added to the simulated timecourses, reflecting natural variations in network connectivity. α, β are scalar parameters which set the contribution of each temporal component. The sampling rate of $m(t)$ was 2 Hz (reflecting the 0.5 s steps in the sliding window used in real data). These network timecourses (averaged across minute long trials) are shown in Figure S1.

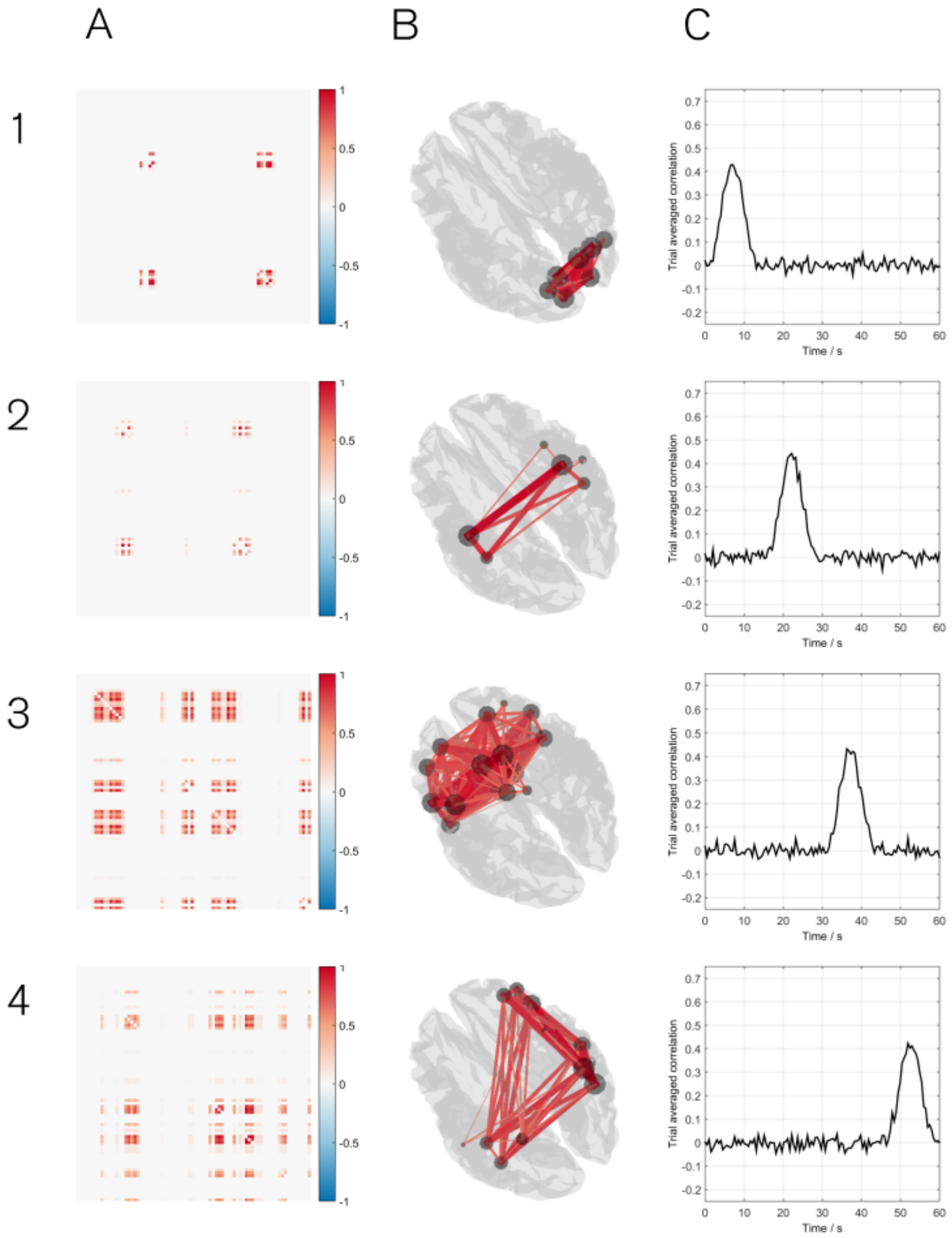

Fig S1: The four simulated networks and their respective connectivity time courses. The separate rows represent 1) visual 2) sensorimotor 3) superior frontal and 4) fronto-parietal networks. The columns represent A) The adjacency matrices of each network, B) A 3D representations of the same network. C) The connectivity timecourse. Here, α = 0.45, β = 0.15.

In order to generate the network contribution, $\boldsymbol{S_{sim}}$, to the simulated adjacency $\boldsymbol{R_{sim}}$, The 4 separate network matrices were combined in a weighted sum, such that $\boldsymbol{S_{simj}}(t)$, the single adjacency matrix at single time point t, is given by:

$$\boldsymbol{S_{sim}}(t) = \sum_{j=1}^{4} m_j(t)\boldsymbol{P}_\mathrm{j} \qquad \text{[S2]}$$

Concatenation over time generated $\boldsymbol{S_{sim}}$, which represents transient formation of our 4 simulated networks over a timecourse spanning 60 minutes.

In order to add realistic interference to our simulated adjacency tensor we added a second adjacency tensor, $\boldsymbol{I_{sim}}$, generated using empty room MEG data. 60 minutes of empty room data were recorded at 600 Hz using a 275-channel MEG system (MISL, Coquitlam, BC, Canada) in synthetic third order gradiometer configuration. Empty room data were processed in the same way as the data in the main paper (save for correcting for leakage) to form $\boldsymbol{I_{sim}}$. Note here that because we do not perform leakage correction, common mode signals recorded at the MEG sensors in the empty room recording will correlate over AAL regions. This manifests as networks of connectivity which will necessarily change in time (according to fluctuating interference). The interference tensor, $\boldsymbol{I_{sim}}$, thus effectively represents networks of no interest. [Note that in reality networks of no interest could be formed through environmental noise, biological interference, or the brain itself. Although we use empty room noise to generate $\boldsymbol{I_{sim}}$, we use this construct to represent all correlations of no interest that would be recoded experimentally.]

The values contained in both $\boldsymbol{S_{sim}}$ and $\boldsymbol{I_{sim}}$ are representative of correlation coefficients (i.e. are scaled between -1 and 1). (I.e. values in $\boldsymbol{S_{sim}}$ represent genuine correlations caused by interactions in brain regions represented by our networks in Figure 1, whereas values in $\boldsymbol{I_{sim}}$ represent artefactual correlations. These two tensors were then combined according to the equation

$$\boldsymbol{R_{sim}} = \frac{S_{sim}}{1+(\frac{1}{SNR})} + \frac{S_{sim}}{1+SNR}, \qquad \text{[S3]}$$

where that SNR represents the effective signal to noise of the data. A derivation showing the origins of this combination of correlation coefficients is given in our appendix below.

Testing the effectiveness of ICA

We applied the ICA decomposition described in the main paper to $\boldsymbol{R_{sim}}$ in order to test whether, in the presence of interference, we would accurately reconstruct the spatial signatures and timecourses of the stimulated networks. The similarities between simulated and reconstructed data were measured by Pearson correlation between 1) simulated and reconstructed timecourses and 2) simulated and reconstructed adjacency matrices. To summarise the performance of ICA in a single run, for a single network, we developed *figure of merit*. This was defined as the correlation of the best matching independent component to a simulated network, minus the mean correlations to all other components. Mathematically, if the ith

independent component timecourse ($\boldsymbol{X}_i$) best matches the jth simulated network timecourse ($\boldsymbol{m}_j$), then the figure of merit would be

$$F_j = r(\boldsymbol{m}_j, \boldsymbol{X}_i) - \frac{1}{n_{ic}-1}\sum_k r(\boldsymbol{m}_j, \boldsymbol{X}_{k\neq i}), \qquad [S4]$$

F can range between 0 and 1, where 1 would correspond to a single independent component representing a network with no other component contributing. Importantly, the figure of merit penalises both poor representation and degeneracy, that is to say when all components can equally explain a single simulated network.

Testing SNR and temporal overlap

We extracted 7 components ($n_{ic} = 7$), and SNR was allowed to range between 0 and 3 in steps of 0.02. For these initial simulations, the onset of the Hanning window was as described above (i.e. on the 3rd, 18th, 33rd and 48th second of every minute for the visual, sensorimotor, frontal and fronto-parietal networks respectively).

We also tested the effect of changing the overlap between temporal representation of networks. In the initial simulation, the time lag between networks peak connectivity was Δ=15 s. However, we wanted to investigate the effect of reducing Δ to the point when two networks timecourses would overlap (i.e. networks were no longer independent). To do this, the simulation was simplified to use only two networks (1 and 2) and the interference removed. Δ was changed from 0 s to 15 s in 1 s steps; ICA was asked to return only 2 components.

Results:

Figure S2 shows the reconstruction of 7 networks, based upon ICA decomposition of the simulated adjacency tensor $\boldsymbol{R}_{sim}$. Panel A shows the reconstructed timecourses whilst Panel B shows the 3D visualisation of the network. It is clear that all four of the simulated networks in Figure S1 have been reconstructed successfully, alongside three separate noise components. The bar plots in panel C show the degree of temporal (left hand plot) and spatial (right hand plot) correlation between independent components and the original simulated networks. I.e. for each reconstructed component, the temporal/spatial correlation with all 4 simulated networks is shown. For both the spatial and temporal plot, a single independent component clearly matches the simulated network. It is noteworthy that these bar charts form the basis of the figure of merit described above.

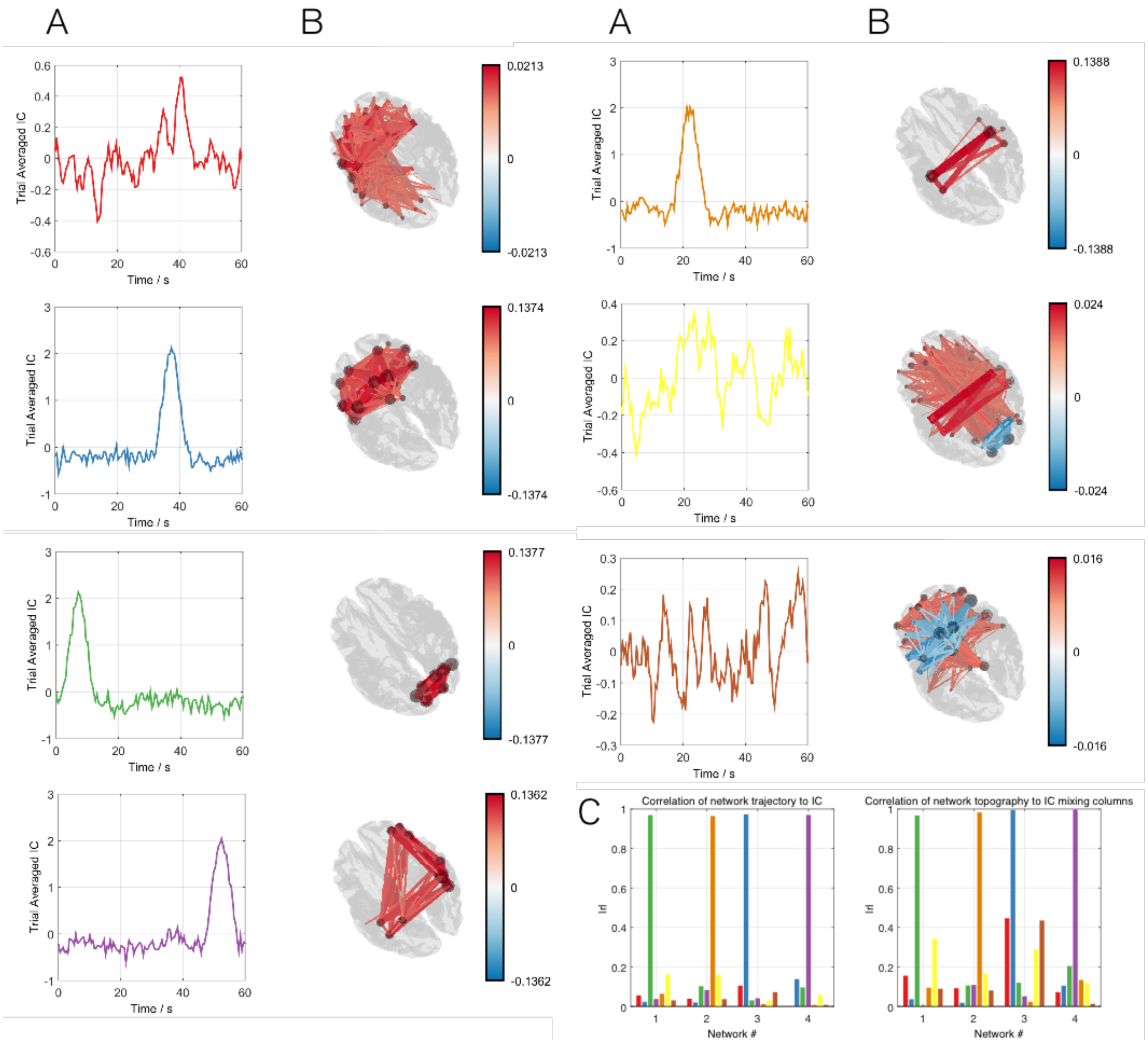

Figure S2: Reconstruction of 7 networks, based upon ICA decomposition of the simulated adjacency tensor $\boldsymbol{R}_{sim}$. A) The trial averaged independent component timecourses. B) 3D representations of the independent component (i.e. corresponding columns of the mixing matrix) thresholded to 50% of the maximum value. C) Bar plots showing temporal (left) and spatial (right) correlation between the reconstructed independent components and the original simulated networks. The columns are colour coded to match the colours of the timecourses, and show there is one clear match, with little residual mixing.

Figure S3 shows our temporal figure of merit plotted against SNR, for all four simulated networks. At high SNR, all 4 simulated networks are reconstructed faithfully using ICA. However, decreasing SNR sees a sharp transition meaning that, below some minimum ‘threshold’ value, a specific network is no longer reconstructed. Importantly, these thresholds differ across networks, showing, for example, that the frontal or fronto-parietal networks can be reconstructed using data at a lower SNR compared to the visual or sensorimotor networks. This interesting dissociation likely results from the number of connections in the network; It can be seen clearly from the matrices in Figure S1 that the visual and sensorimotor networks have less connections than the frontal and fronto-parietal networks, and it is these networks that require higher SNR. This relationship is further shown by the graph in Figure S3B. Here, number of connections is represented by the Frobenius norm of the network adjacency matrix. Note that Frobenius norm (given by $\|\boldsymbol{A}\|_F = \sqrt{\sum_i \sum_j a_{ij}^2}$ where a_{ij} represents

the element in the ith row and j[th] column of $\boldsymbol{A}$) usually represents both number and strength of connections, however since maximum strength of any connection (maximum value of a_{ij}) in each simulated matrix was made equal, Frobenius norm, in this case, is solely representative of spatial extent. Figure S3B shows clearly that SNR is a monotonically decreasing function of norm, meaning that networks with large extent can be reconstructed using lower SNR data.

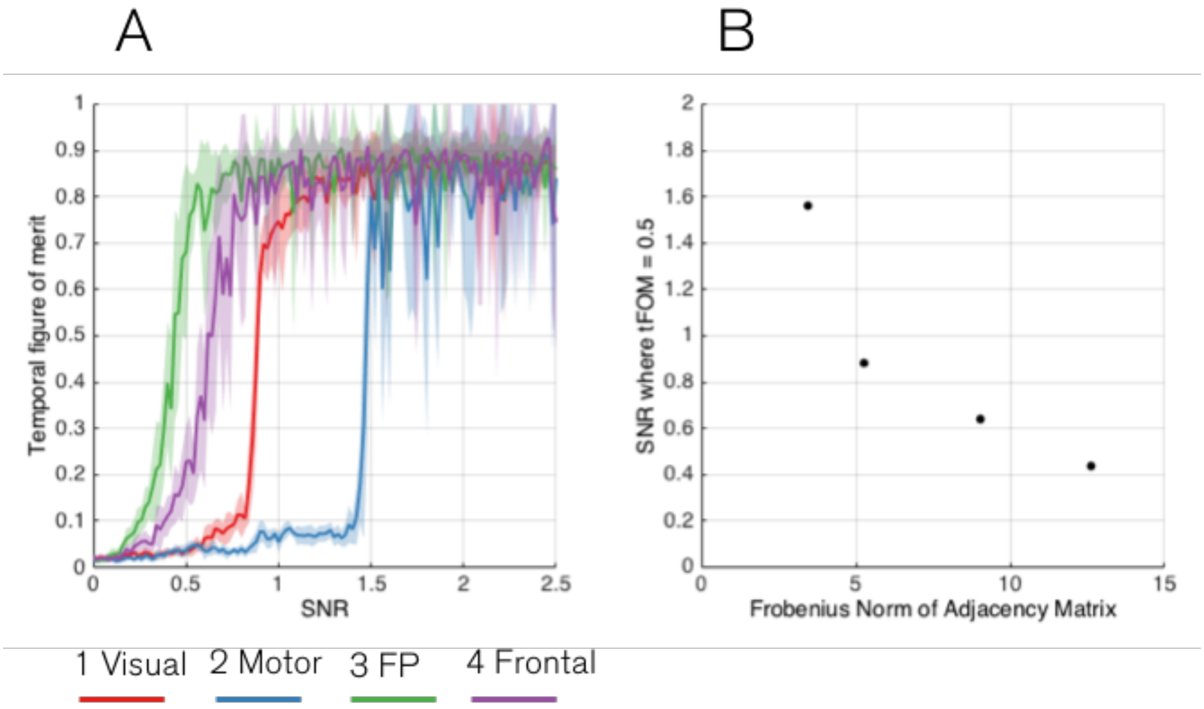

Figure S3: The effect of the signal to noise ratio (SNR). A) The mean temporal figure of merit for each network plotted as a function of SNR; note that for high SNR all networks are well characterised. However, a critical SNR exists below which our ability to successfully reconstruct any one network drops. Panel B shows the relationship between the Frobenius Norm of the simulated adjacency matrix (representing spatial extent of the network) and critical SNR (defined as the SNR required for a temporal figure of merit of 0.5). Note that adjacency matrices with a high Frobenius norm can be reconstructed using lower SNR data.

Finally, Figure S4 shows the effect of increasing the temporal overlap between simulated network timecourses (i.e. decreasing Δ). It is well known that ICA attempts to pull out temporally independent timecourses meaning that, when timecourses of separate networks begin to overlap, the spatial representations of those networks will become mixed. This is a fundamental assumption of ICA, and thus the way in which networks are characterised using our method. Figure S4A shows the temporal Figure of merit (for a 2 network simulation, see above) plotted as a function of Δ. As expected when timecourses become mixed, the networks are no longer reconstructed separately. Panels B, C and D show both temporal and spatial representations of the independent components for $\Delta = 6$ s, $\Delta = 3$ s and $\Delta = 0$ s respectively. As expected, with no temporal overlap ($\Delta = 6$ s) both the spatial and temporal signature of the two extracted components represent a single simulated network. However, with increasing overlap those spatial and temporal properties combine, meaning that the two networks are no longer separable.

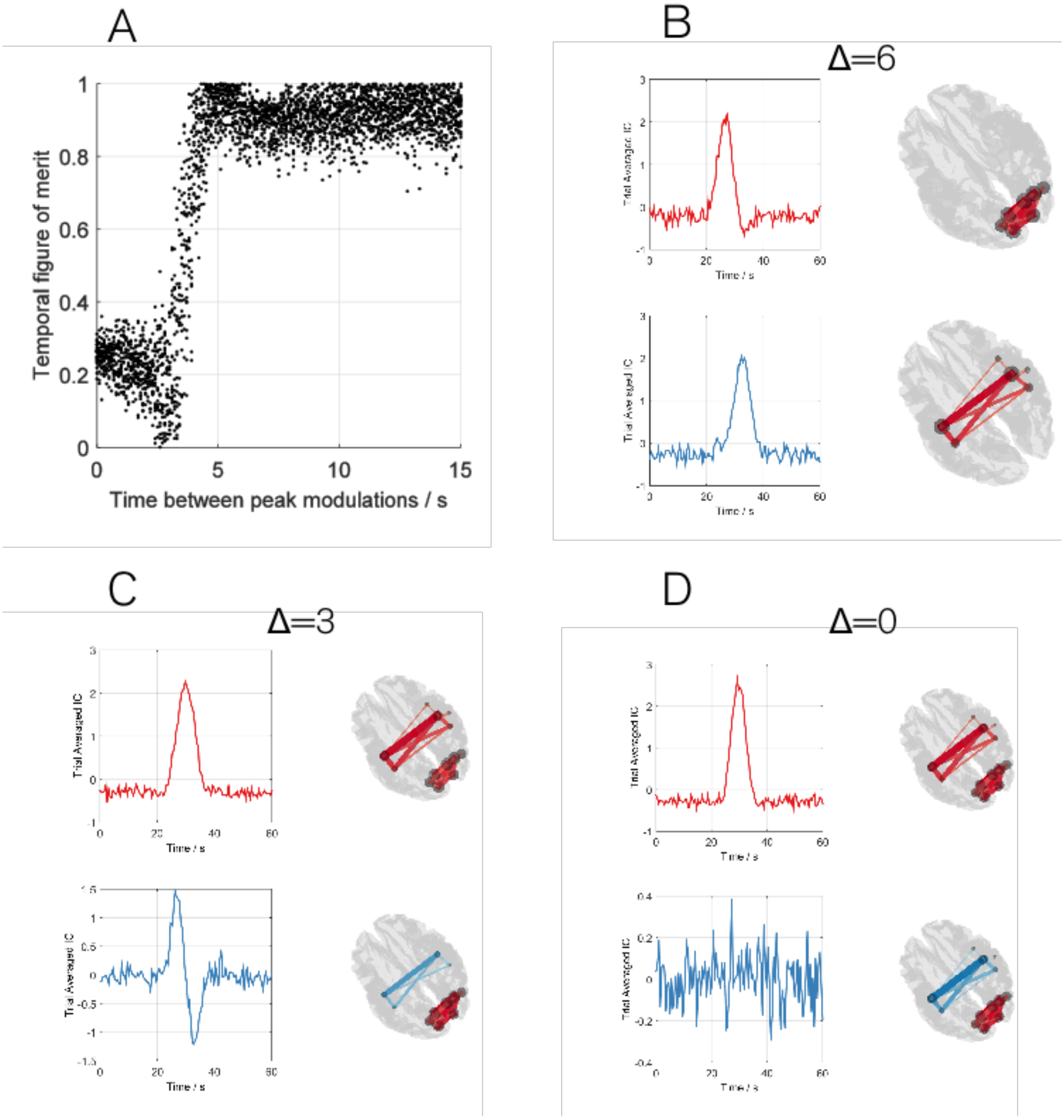

Figure S4: Effect of reducing the temporal independence between networks. A) Temporal Figure of merit plotted as a function of Δ. Note that, as would be expected, when timecourses become mixed the networks are no longer faithfully reconstructed. Panels B)-D) show the independent components and their corresponding network topographies for a range of Δ values. Note that network topographies in blue represent connections whose timecourses are anticorrelated with the timecourse shown.

Discussion

We have shown in simulation that ICA can successfully separate functional networks based on their connectivity timecourses. However, importantly, we show that success of this algorithm is a function of both signal to noise ratio and the number of connections in a network. As would be expected, the ability of ICA to uniquely extract networks is diminished at low signal to noise ratio. However, the point of failure (minimum SNR at which the network is accurately reconstructed) varies as a function of the number of connections, with spatially extended networks being more robustly detectable at low SNR. This important point should be

considered in all future uses of this or related methodologies. In addition, as would be expected with an ICA based method, the reconstruction fails when network timecourses are no longer temporally orthogonal.

References

Brookes, M.J., O'Neill, G.C., Hall, E.L, Woolrich, M.W, Baker, A., Palazzo Corner, S., Robson, S.E., Morris, P.G., Barnes, G.R, 2014. Measuring temporal, spectral and spatial changes in electrophysiological brain network connectivity. NeuroImage 91, 282-99.

Brookes, M.J., Hall, E.L., Robson, S.E., Price, D., Palaniyappan, L., Liddle, E.B., Liddle, P.F., Robinson, S.E., Morris, P.G., 2015. Complexity measures in magnetoencephalography: measuring "disorder" in schizophrenia. PLoS One 10, e0120991.

Gong, G., He, Y., Concha, L., Lebel, C., Gross, D.W., Evans, A.C., Beaulieu, C., 2009. Mapping anatomical connectivity patterns of human cerebral cortex using in vivo diffusion tensor imaging tractography. Cereb Cortex 19, 524-536.

Huang, M.X., Mosher, J.C., Leahy, R.M., 1999. A sensor-weighted overlapping-sphere head model and exhaustive head model comparison for MEG. Phys Med Biol 44, 423-440.

Hyvarinen, A., 1999. Fast and robust fixed-point algorithms for independent component analysis. IEEE Trans Neural Netw 10, 626-634.

O'Neill, G.C., Bauer, M., Woolrich, M.W., Morris, P.G., Barnes, G.R., Brookes, M.J., 2015. Dynamic recruitment of resting state sub-networks. NeuroImage 115, 85-95.

Robinson, S., Vrba, J., 1999. Functional neuroimaginf by synthetic aperture magnetometry (SAM). In: Yoshimoto, T., Kotani, M., Kuriki, S., Karibe, H., Nakasato, N. (Eds.), Recent advances in biomagnetism. Tohoku University Press, Sendai, pp. 302-305.

Sarvas, J., 1987. Basic mathematical and electromagnetic concepts of the biomagnetic inverse problem. Phys Med Biol 32, 11-22.

Appendix A: Derivation of Equation S3

The correlation coefficient between two vectors, $\boldsymbol{a}$ and $\boldsymbol{b}$ is given by

$$r = \frac{\boldsymbol{a}\boldsymbol{b}^T}{\sqrt{(\boldsymbol{a}\boldsymbol{a}^T)(\boldsymbol{b}\boldsymbol{b}^T)}}. \quad \text{[A1]}$$

This value is bounded between -1 and 1. Note that the values $\boldsymbol{a}\boldsymbol{a}^T$ and $\boldsymbol{b}\boldsymbol{b}^T$ reflect the un-normalised variance of $\boldsymbol{a}$ and $\boldsymbol{b}$.

Now, let us assume that $\boldsymbol{a}$ and $\boldsymbol{b}$ are each made up of two signals, a signal of interest representing real brain activity and a signal representing a source of interference.

$$\boldsymbol{a} = \boldsymbol{a}_s + \boldsymbol{a}_i \quad \text{[A2]}$$
$$\boldsymbol{b} = \boldsymbol{b}_s + \boldsymbol{b}_i \quad \text{[A3]}$$

For our simulations, $\boldsymbol{a}_s$ and $\boldsymbol{b}_s$ reflect genuine network connectivity (i.e. they will generate the network adjacency matrices) and $\boldsymbol{a}_i$ and $\boldsymbol{b}_i$ represent the noise. So, when an adjacency matrix for a given network is defined in $\boldsymbol{S}_{sim}$, the elements reflect

$$r_s = \frac{\boldsymbol{a}_s\boldsymbol{b}_s^T}{\sqrt{(\boldsymbol{a}_s\boldsymbol{a}_s^T)(\boldsymbol{b}_s\boldsymbol{b}_s^T)}}. \quad \text{[A4]}$$

Likewise, the adjacency matricies from the empty room noise in $\boldsymbol{I}_{sim}$ contain elements represented by

$$r_i = \frac{\boldsymbol{a}_i\boldsymbol{b}_i^T}{\sqrt{(\boldsymbol{a}_i\boldsymbol{a}_i^T)(\boldsymbol{b}_i\boldsymbol{b}_i^T)}}. \quad \text{[A5]}$$

In order to simulate realistic connectivity matrices containing both signal and noise (i.e $\boldsymbol{R}_{sim}$), we could just add r_s and r_i, however this may result in simulated correlation values with a magnitude larger than 1. For this reason we need to examine carefully how to combine them. The true combined correlation coefficient is

$$r = \frac{(\boldsymbol{a}_s+\boldsymbol{a}_i)(\boldsymbol{b}_s+\boldsymbol{b}_i)^T}{\left((\boldsymbol{a}_s+\boldsymbol{a}_i)(\boldsymbol{a}_s+\boldsymbol{a}_i)^T(\boldsymbol{b}_s+\boldsymbol{b}_i)(\boldsymbol{b}_s+\boldsymbol{b}_i)^T\right)^{0.5}}, \quad \text{[A6]}$$

which is equal to

$$r = \frac{\boldsymbol{a}_s\boldsymbol{b}_s^T+\boldsymbol{a}_s\boldsymbol{b}_i^T+\boldsymbol{a}_i\boldsymbol{b}_s^T+\boldsymbol{a}_i\boldsymbol{b}_i^T}{\left((\boldsymbol{a}_s\boldsymbol{a}_s^T+\boldsymbol{a}_s\boldsymbol{a}_i^T+\boldsymbol{a}_i\boldsymbol{a}_s^T+\boldsymbol{a}_i\boldsymbol{a}_i^T)(\boldsymbol{b}_s\boldsymbol{b}_s^T+\boldsymbol{b}_s\boldsymbol{b}_i^T+\boldsymbol{b}_i\boldsymbol{b}_s^T+\boldsymbol{b}_i\boldsymbol{b}_i^T)\right)^{0.5}} \quad \text{[A7]}$$

since $\boldsymbol{a}_s$ represents signal and $\boldsymbol{a}_i$ represents noise, we can assume that the signal and noise are uncorrelated, and so $\boldsymbol{a}_s\boldsymbol{a}_i^T = 0$ and $\boldsymbol{b}_s\boldsymbol{b}_i^T = 0$. We also allow the interference to be correlated between regions and likewise the signal to be correlated between regions, meaning $\boldsymbol{a}_s\boldsymbol{b}_s^T$ and $\boldsymbol{a}_i\boldsymbol{b}_i^T$ are finite. However we assume that the signal at region 1 ($\boldsymbol{a}_s$) does not correlate with interference at region 2 ($\boldsymbol{b}_i$) and vice versa. Therefore, $\boldsymbol{a}_s\boldsymbol{b}_i^T = 0$ and $\boldsymbol{b}_s\boldsymbol{a}_i^T = 0$ meaning Equation A7 can be simplified

$$r = \frac{\boldsymbol{a}_s\boldsymbol{b}_s^T+\boldsymbol{a}_i\boldsymbol{b}_i^T}{\left((\boldsymbol{a}_s\boldsymbol{a}_s^T+\boldsymbol{a}_i\boldsymbol{a}_i^T)(\boldsymbol{b}_s\boldsymbol{b}_s^T+\boldsymbol{b}_i\boldsymbol{b}_i^T)\right)^{0.5}}. \quad \text{[A8]}$$

In the denominator of Equation A8, each of the 4 terms represent variances:

- $\boldsymbol{a}_s\boldsymbol{a}_s^T = v_{as}$, the variance of the signal at region 1;
- $\boldsymbol{a}_i\boldsymbol{a}_i^T = v_{ai}$, the variance of the interference at region 1;
- $\boldsymbol{b}_s\boldsymbol{b}_s^T = v_{bs}$, the variance of the signal at region 2;
- $\boldsymbol{b}_i\boldsymbol{b}_i^T = v_{bi}$, the variance of the interference at region 2.

Therefore:

$$r = \frac{\boldsymbol{a_s b_s^T} + \boldsymbol{a_i b_i^T}}{\left((v_{as}+v_{ai})(v_{bs}+v_{bi})\right)^{0.5}} . \qquad \text{[A9]}$$

We could then assume that the magnitude of the signal and interference is approximately the same across spatially separate regions, i.e:

$$v_{as} = v_{bs} = v_s \qquad \text{[A10]}$$
$$v_{ai} = v_{bi} = v_{\square} \qquad \text{[A11]}$$

therefore,

$$r = \frac{\boldsymbol{a_s b_s^T} + \boldsymbol{a_i b_i^T}}{\left((v_s+v_i)(v_s+v_i)\right)^{0.5}} = \frac{\boldsymbol{a_s b_s^T} + \boldsymbol{a_i b_i^T}}{v_s+v_i} . \qquad \text{[A12]}$$

Now, v_s and v_i which represent the variance of the signal and noise respectively are related by the signal to noise ratio (SNR)

$$v_s = SNRv_i \qquad \text{[A13]}$$

so we can write

$$r = \frac{\boldsymbol{a_s b_s^T}}{v_s + \frac{v_s}{SNR}} + \frac{\boldsymbol{a_i b_i^T}}{SNRv_i + v_i} \qquad \text{[A14]}$$

$$r = \frac{\boldsymbol{a_s b_s^T}}{v_s\left(1+\frac{1}{SNR}\right)} + \frac{\boldsymbol{a_i b_i^T}}{1+SNR} . \qquad \text{[A15]}$$

Returning to Equation A4, note that under our assumptions

$$r_s = \frac{\boldsymbol{a_s b_s^T}}{v_s}, \qquad \text{[A16]}$$

similarly,

$$r_i = \frac{\boldsymbol{a_i b_i^T}}{v_i}, \qquad \text{[A17]}$$

we finally arrive at the expression

$$r = \frac{r_s}{1+\frac{1}{SNR}} + \frac{r_i}{1+SNR} . \qquad \text{[A18]}$$

So this is how the signal and noise adjacency matrices should be combined. Note that for an SNR of 1, you simply average the 2 correlation coefficients, i.e $r = 0.5(r_s + r_i)$, and the value is always bounded by -1 and 1. For extremely high SNR, the term $\frac{r_i}{1+SNR} \rightarrow 0$ and is dominated by the signal. For extremely low SNR (SNR << 1), the term $\frac{r_s}{1+\frac{1}{SNR}} \rightarrow 0$ and we are dominated by interference.

Appendix B: Additional Self Paced Motor Results

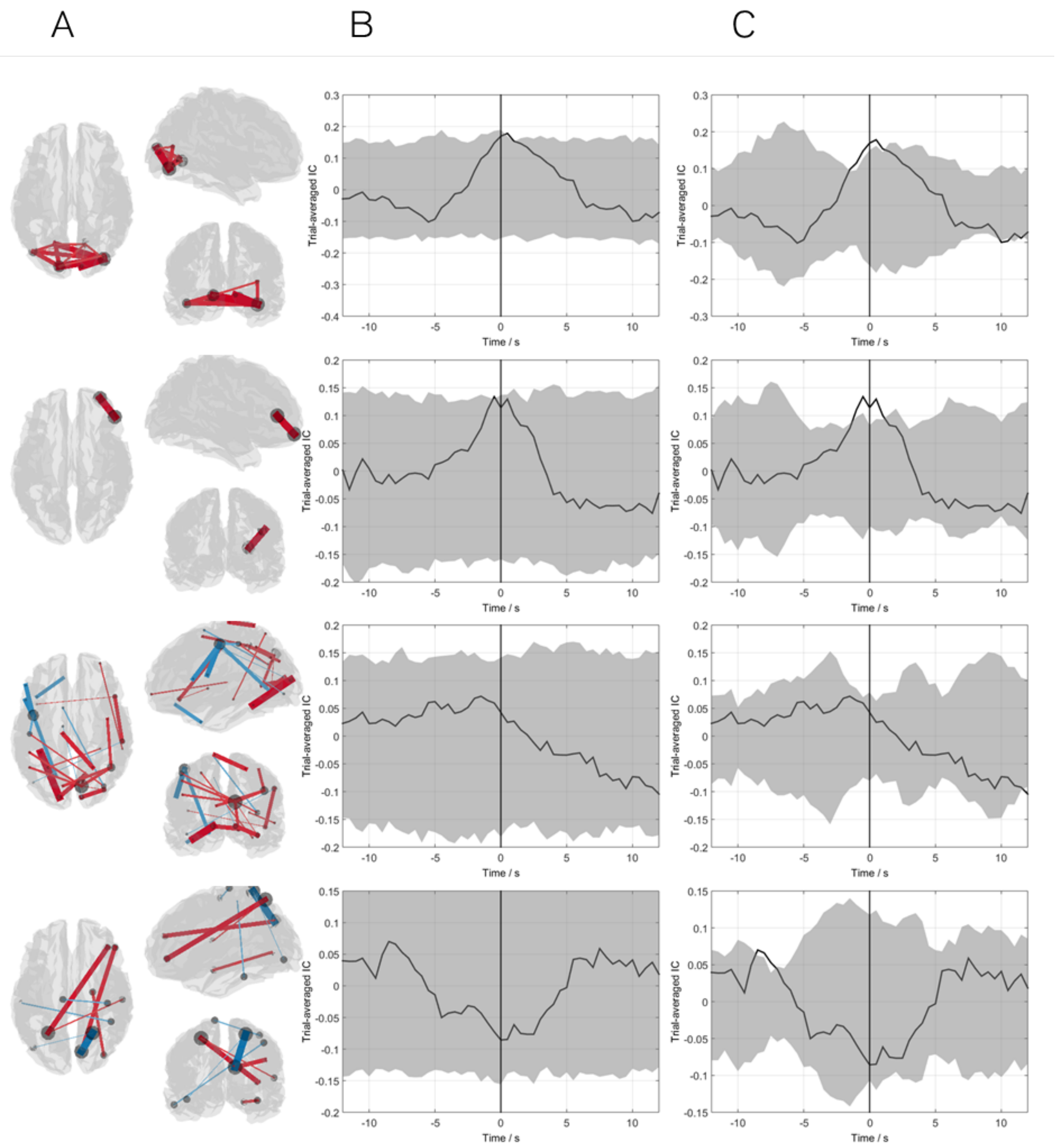

Figure A1: Additional self paced motor results (1-4). Panel A shows the 3D representation of each network derived, with the trial averaged timecourses shown in panels B and C. Panel B shows the null distribution (grey shaded region) based on no significant trial-averaged modulations in connectivity and Panel C's shaded region is the null distribution generated from the sign-flip analysis.

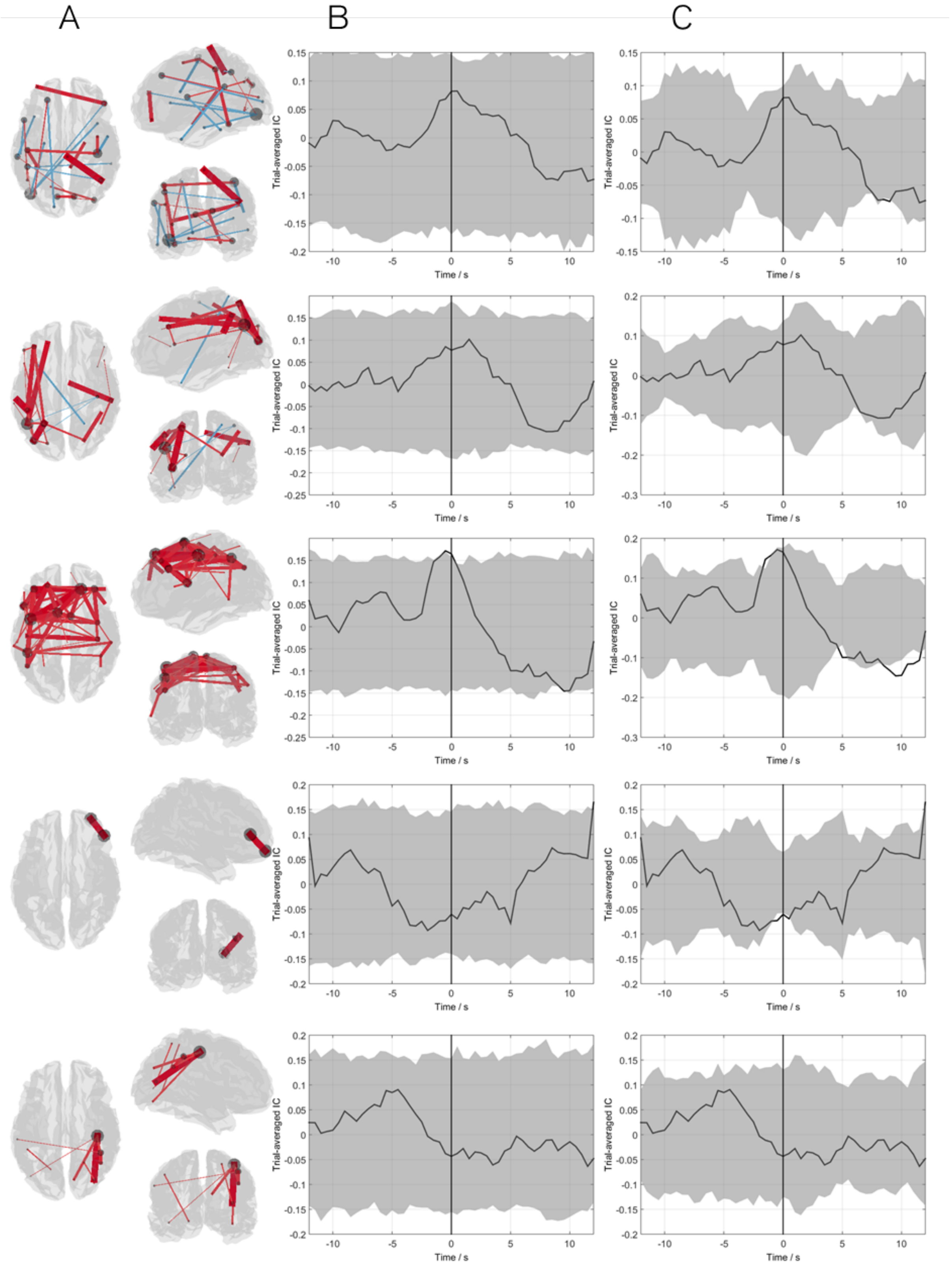

Figure A2: Additional self paced motor results (5-9). Panel A shows the 3D representation of each network derived, with the trial averaged timecourses shown in panels B and C. Panel B shows the null distribution (grey shaded region) based on no significant trial-averaged modulations in connectivity and Panel C's shaded region is the null distribution generated from the sign-flip analysis.
